# Is disturbed clearance of apoptotic keratinocytes responsible for UVB-induced inflammatory skin lesions in systemic lupus erythematosus?

**DOI:** 10.1186/ar2051

**Published:** 2006-10-02

**Authors:** Esther Reefman, Marcelus CJM de Jong, Hilde Kuiper, Marcel F Jonkman, Pieter C Limburg, Cees GM Kallenberg, Marc Bijl

**Affiliations:** 1 Department of Rheumatology and Clinical Immunology, University Medical Center Groningen, University of Groningen, PO Box 30.001, 9700 RB Groningen, The Netherlands; 2 Department of Dermatology, University Medical Center Groningen, University of Groningen, PO Box 30.001, 9700 RB Groningen, The Netherlands; 3 Department of Pathology and Laboratory Medicine, University Medical Center Groningen, University of Groningen, PO Box 30.001, 9700 RB Groningen, The Netherlands

## Abstract

Apoptotic cells are thought to play an essential role in the pathogenesis of systemic lupus erythematosus (SLE). We hypothesise that delayed or altered clearance of apoptotic cells after UV irradiation will lead to inflammation in the skin of SLE patients. Fifteen SLE patients and 13 controls were irradiated with two minimal erythemal doses (MEDs) of ultraviolet B light (UVB). Subsequently, skin biopsies were analysed (immuno)histologically, over 10 days, for numbers of apoptotic cells, T cells, macrophages, and deposition of immunoglobulin and complement. Additionally, to compare results with cutaneous lesions of SLE patients, 20 biopsies of lupus erythematosus (LE) skin lesions were analysed morphologically for apoptotic cells and infiltrate. Clearance rate of apoptotic cells after irradiation did not differ between patients and controls. Influx of macrophages in dermal and epidermal layers was significantly increased in patients compared with controls. Five out of 15 patients developed a dermal infiltrate that was associated with increased epidermal influx of T cells and macrophages but not with numbers of apoptotic cells or epidermal deposition of immunoglobulins. Macrophages were ingesting multiple apoptotic bodies. Inflammatory lesions in these patients were localised near accumulations of apoptotic keratinocytes similar as was seen in the majority of LE skin lesions. *In vivo *clearance rate of apoptotic cells is comparable between SLE patients and controls. However, the presence of inflammatory lesions in the vicinity of apoptotic cells, as observed both in UVB-induced and in LE skin lesions in SLE patients, suggests that these lesions result from an inflammatory clearance of apoptotic cells.

## Introduction

Systemic lupus erythematosus (SLE) is a systemic autoimmune disease characterised by the presence of autoantibodies directed against nuclear and cytoplasmic antigens in combination with a wide range of clinical manifestations. Photosensitivity is one of its manifestations, affecting 30% to 50% of patients [1-3]. Most cutaneous lupus lesions can be triggered by sunlight exposure. Sunlight exposure, especially ultraviolet B light (UVB), can even induce systemic disease activity. UVB is a potent inducer of apoptosis. During the last decade, it has become clear that apoptotic cells play an important role in autoimmunity, in particular SLE [4].

During the process of apoptosis, intracellular antigens are expressed on the surface of the apoptotic cell and exposed to the immune system [5]. In susceptible mice and rats, injection of apoptotic cells results in loss of tolerance, autoantibody formation, and even clinical disease [6,7]. In humans, the role of apoptotic cells in the induction of autoimmunity is not yet clear. In established SLE, decreased clearance of apoptotic cells by macrophages [8-10], increased levels of circulating apoptotic cells [11,12], and presence of apoptotic cells in lupus skin lesions [13] have been reported. Whether accumulation of apoptotic cells induces autoimmunity and/or drives the autoimmune disease after tolerance has been broken, has not yet been elucidated.

Apoptotic epidermal cells can be recognised in the skin by their pyknotic nuclei and eosinophilic cytoplasm in sections stained with haematoxylin eosin (H&E) and are known as sunburn cells (SBCs) [14]. SBCs can be detected as early as 8 hours after UVB exposure, with maximal numbers being present at 24 to 48 hours [15]. We previously showed that induction of SBCs in the skin of patients with SLE does not differ from that in healthy controls after a single standardised dose of UVB [16].

Apoptotic cells are formed in several tissues as part of normal tissue homeostasis or are induced by influences from the environment. Under physiological circumstances, phagocytes can rapidly clear apoptotic cells without causing any tissue damage. Upon ingestion of apoptotic cells, phagocytes release anti-inflammatory cytokines such as transforming growth factor-β. In patients with SLE, however, autoantibodies may recognise autoantigens exposed on the surface of apoptotic cells [5]. Binding of autoantibodies to apoptotic cells can result in Fcγ-receptor (FcγR)-mediated clearance of apoptotic cells. It is conceivable that this leads to inflammation given that ligation of FcγR induces the release of pro-inflammatory cytokines [17,18]. In this study, we analysed whether apoptotic keratinocytes in patients with SLE, as induced by a single dose of UVB, are cleared with delay and/or in an inflammatory way that results in the development of inflammatory skin lesions.

## Materials and methods

### Patients and controls

Patients eligible for the study fulfilled at least four ACR (American College of Rheumatology) criteria for SLE [19] and had inactive disease, defined as SLEDAI (SLE Disease Activity Index) of not greater than 4. Patients with active skin disease, and those patients and controls whose buttock skin had been exposed to sunlight or other sources of UVB in the past 6 months, were excluded from the study. The local ethics committee of the University Medical Center Groningen (The Netherlands) approved the study, and all patients and controls gave written informed consent according to the Declaration of Helsinki. Fifteen patients (age 48.7 ± 12.3 years [mean ± standard deviation (SD)]; four males, 11 females) were included. Skin types were determined based on the Fitzpatrick skin typing chart [20]. Skin type distribution (apart from two type 5 or higher scores in non-Caucasian patients) was similar in patients (three type 2, 10 type 3, one type 5, and one type 6) and controls (four type 2 and nine type 3). Table 1 shows patient characteristics and immunosuppressive medication used at the time of the study. Autoantibodies to double-stranded DNA were measured by Farr assay, and antibodies to SSA/Ro, SSB/La, nRNP, and Sm were assessed by counter immuno-electrophoresis. Anti-cardiolipin antibodies, immunoglobulin (Ig) G or IgM, were tested by enzyme-linked immunosorbent assay. Minimal erythemal dose (MED) was determined as described below. Thirteen healthy volunteers (age 33.8 ± 15.8 years [mean ± SD]; five males, eight females) were included as controls. Additionally, to compare results with established cutaneous lesions of patients with SLE, 20 biopsies of LE skin lesions were retrieved from the files of the Department of Pathology (Table 2).

### Irradiation protocol

UVB irradiation was performed using the Waldman 800 'sky' lights with TL-12 lamps (Philips, Eindhoven, The Netherlands) at a distance of 15 cm from the buttock skin. A Diffey grid [21] was used to irradiate the skin with 10 different doses (0.026 to 0.200 J/cm^2^) during one exposure. After 24 hours, MED was determined by two independent observers, with more than 90% agreement. MED is defined as the lowest UVB dose at which erythema can be detected in the skin. In case of disagreement between observers, the mean of the two values was used. The reproducibility of MED assessment was determined by a second irradiation in 10 subjects, five healthy controls and five patients. Inter-test variability ranged from 0% to 22% (median 3%). After assessment of MED, subjects were irradiated with two MEDs of UVB on four small areas (1 × 2.5 cm/area) on the other buttock. After 1, 3, and 10 days, 4 mm skin biopsies were taken from the area of skin irradiated with two MEDs. As a control, a biopsy was taken after 1 day from non-irradiated skin. The distance between the biopsies was at least 2 cm to avoid the influence of wound healing on the reaction to UVB. Biopsies were split, one half fixed in formaldehyde and the other half snap-frozen in liquid nitrogen.

### Chemicals and antibodies

Diaminobenzidine (DAB) solution contained 25 mg DAB and 50 mg imidazol in 50 ml phosphate-buffered saline (PBS) and was filtrated before use. AEC (3-amino-9-ethylcarbazole). (Sigma-Aldrich, St. Louis, MO, USA) stock solution was diluted 100× in acetate buffer (0.05 M, pH 5.0). To both staining solutions, H_2_0_2 _was added just before incubation of the sections, resulting in a concentration of 0.1% H_2_0_2_. For haematoxylin staining, Mayer's haemalum solution (Merck, Darmstadt, Germany) was used.

Rabbit antibodies to cleaved caspase-3 (no. 9661S) were purchased from Cell Signaling Technology, Inc. (Danvers, MA, USA). Primary fluorescein isothiocyanate (FITC)-labeled goat F(ab)_2 _antibodies directed against human IgM, IgA, and IgG were purchased from Protos Immunoresearch (Burlingame, CA, USA). Primary mouse antibodies directed against CD68 (clone PGGM 1), CD3-FITC-labeled (clone F7.2.38), C3c-FITC-labeled (clone F201), and C1q-FITC labeled (F0254), and all secondary antibodies (that is, goat anti-rabbit IgG-horseradish peroxidase [HRP], rabbit anti-goat IgG-HRP, and rabbit anti-mouse IgG-HRP) were obtained from DakoCytomation (Glostrup, Denmark).

### Immunohistochemistry

Skin sections (4 μm) on APES (3-amino-propyltriethoxysilane)-coated glass slides were used for all experiments. Sections were deparaffinised by subsequent incubations in xylene (10 minutes), 100% ethanol (5 minutes), and 96% ethanol (2 minutes), twice, followed by ethanol 70% (2 minutes) and distilled water. H&E staining was performed according to standard protocol using the linear stainer from medite Medizintechnik GmbH (Burgdorf, Germany). Antigen retrieval was performed by boiling in 1 mM EDTA (ethylenediaminetetraacetic acid) pH 8.0 (cleaved-caspase-3 staining), 10 mM citrate pH 6.0 (CD68 staining), or 10 mM Tris, 1 mM EDTA pH 9.0 (CD3 staining). After washing in PBS, endogenous peroxidase was blocked by incubation in 0.37% H_2_0_2 _in PBS for 30 minutes. Slides were incubated with various antibodies (diluted 1:75 for anti-cleaved caspase-3 and 1:50 for CD68 and CD3, in 1% bovine serum albumin [BSA]/PBS) for 1 to 2 hours at room temperature. Subsequently, slides were washed in PBS (three times) and incubated for 30 minutes at room temperature with either goat anti-rabbit IgG-HRP (for cleaved caspase-3 staining) or rabbit anti-mouse IgG-HRP (for CD68 and CD3 staining) (1:50 in 1% BSA/PBS) and then washed again (three times) in PBS followed by incubation with rabbit anti-goat IgG-HRP or goat anti-rabbit IgG-HRP, respectively, for another 30 minutes at room temperature. After washing in PBS (three times), slides were incubated in DAB solution for 15 to 20 minutes and subsequently washed with distilled water (five times). Slides were then counterstained with haematoxylin for 1 minute, washed in distilled water (five times), dehydrated in 96% ethanol and subsequently in 100% ethanol, and then mounted. Using Leica QWin software (Leica Microsystems, Cambridge, UK), morphometry was performed on entire skin tissue sections stained with antibodies against CD68 or CD3. Epidermal and papillary dermal layers (approximately 150 μm of dermal layer directly localised beneath the epidermis) were assessed separately by manually drawing a line around these layers under ×100 magnification.

### Scoring procedure for apoptotic cells

**Figure 1:**
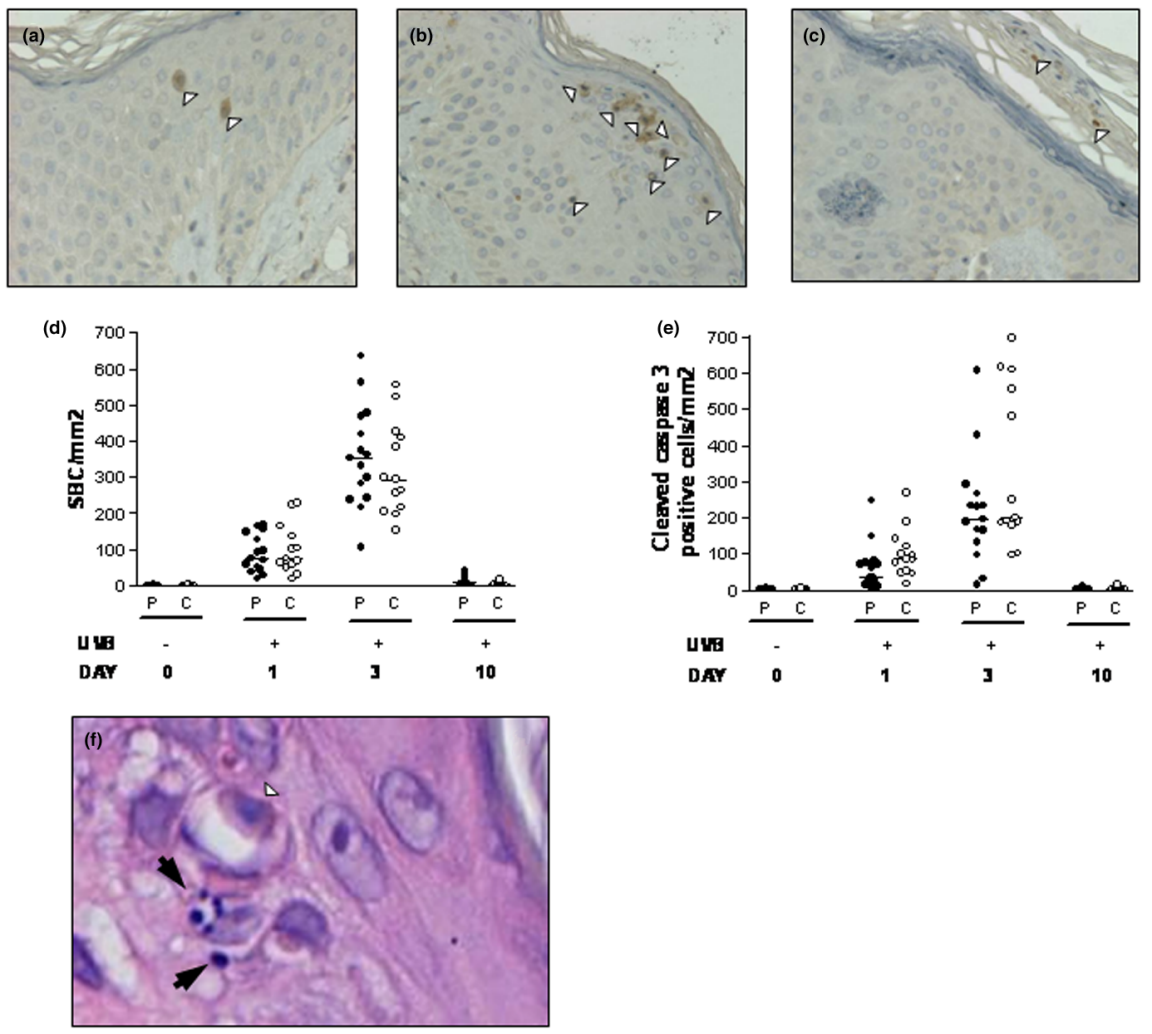
Clearance of apoptotic cells from irradiated skin in patients with systemic lupus erythematosus (SLE) compared with controls. Representative images of skin sections stained for cleaved caspase-3 **(a) **1 day, **(b) **3 days, and **(c) **10 days after irradiation with two minimal erythemal doses of ultraviolet B light (UVB). Arrowheads indicate cleaved caspase-3-positive cells. Magnifications, ×200. **(d) **Graph showing numbers of sunburn cells (SBCs) per square millimeter in patients (*n *= 15) and controls (*n *= 13). **(e) **Number of cleaved caspase-3-positive keratinocytes per square millimeter. ●, patients (P); ○, controls (C). Median is indicated by a horizontal line. **(f) **A representative hematoxylin eosin (H&E)-stained section after irradiation with UVB, showing SBCs (white arrowheads) and nuclear dust (black arrows indicate one intact pyknotic nucleus, white arrow indicates pyknotic fragmented nucleus).

Using Olympus Soft Pro software (Tokyo, Japan), the surface area of the epidermis was determined by manually drawing a line around this area and calculating the total surface (mm^2^). Subsequently, numbers of SBCs and nuclear dust were scored in three sequential H&E-stained sections. Nuclear dust was defined as one whole pyknotic nucleus or a group of pyknotic nuclear fragments (as indicated in Figure 1f by black and white arrows, respectively). Numbers of SBCs or extent of nuclear dust per square millimeter was determined by dividing the counted numbers by the epidermal surface area and calculating the mean value of the three sections. Cleaved caspase-3-positive cells were scored accordingly.

### Scoring procedure for infiltrating cells and inflammatory lesions

Infiltrating cells in the dermis were scored semi-quantitatively and by morphometry. H&E sections were semi-quantitatively scored for the presence of infiltrating cells using a score from 0 to 5. In short, vessels in the papillary dermis were scored blindly for the presence of perivascular infiltrating cells, in three consecutive sections: no infiltrating cells (0), not more than two infiltrating cells (1), not more than one perivascular layer of infiltrating cells (2), two or three layers of infiltrating cells (3), more than three layers of infiltrating cells (4), and more than three layers of infiltrating cells in combination with clear progression outside the perivascular region (5). The final score was determined by averaging the mean vessel score of three consecutive sections. Morphometry was performed on the dermal layer of CD3- and CD68-stained sections. Infiltrates in patients were considered present when the semi-quantitative scores of dermal influx of infiltrating cells in H&E-stained sections and the morphometric scores of either CD3- or CD68-stained OK sections were increased (> mean + two SDs of controls) in biopsies taken on at least two different days, after UVB irradiation.

**Figure 2:**
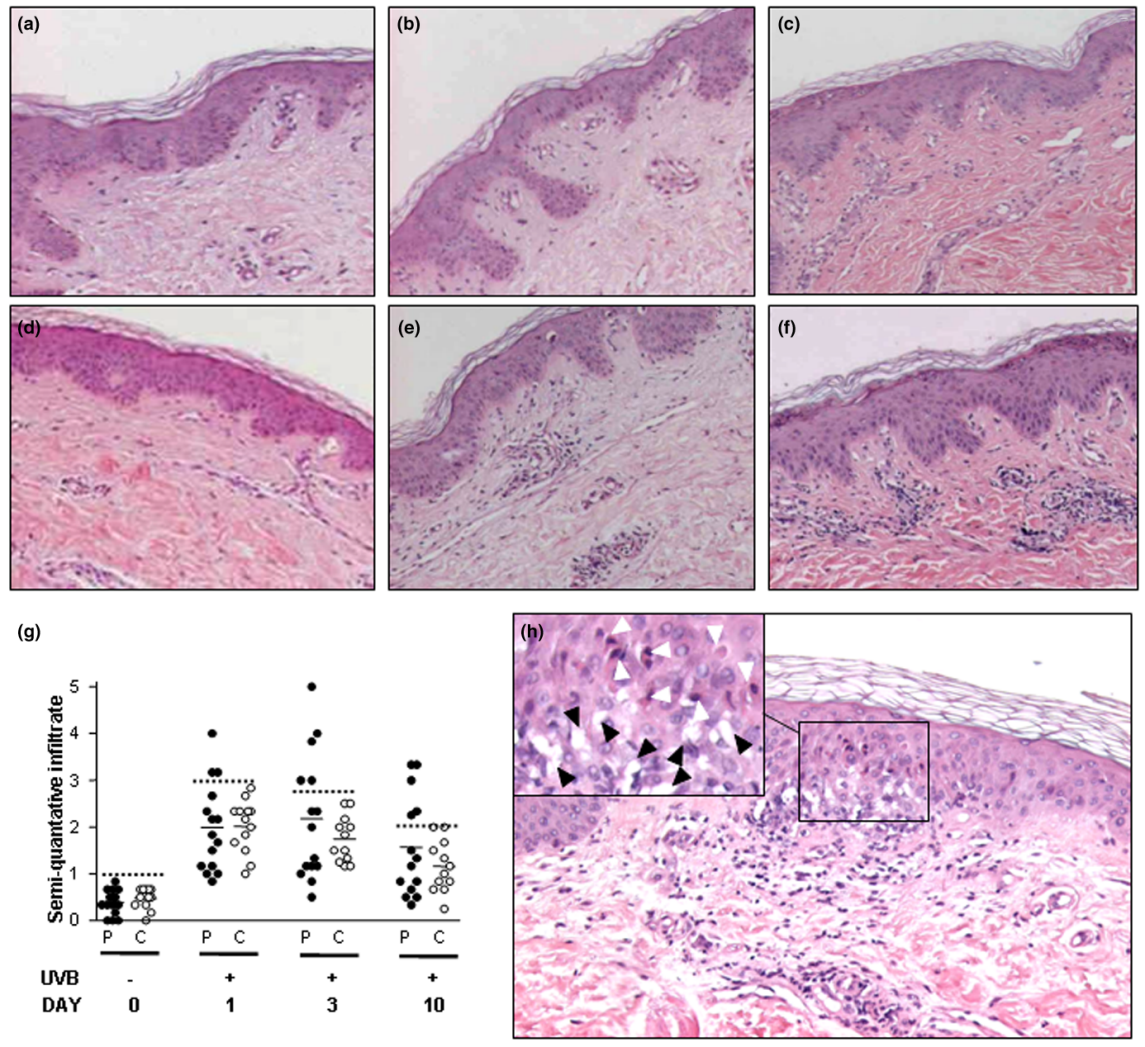
Development of infiltrates and inflammatory lesions in the vicinity of sunburn cells (SBCs) in patients with systemic lupus erythematosus (SLE). Haematoxylin eosin (H&E)-stained paraffin sections before and after irradiation with two minimal erythemal doses of ultraviolet B light (UVB). **(a-c) **Biopsies from a representative control, non-irradiated **(a) **and 1 **(b) **and 3 **(c) **days after irradiation. **(d-f) **Biopsies from a representative patient with increased influx of cells, non-irradiated **(d) **and 1 **(e) **and 3 **(f) **days after irradiation. Magnifications, ×100. **(g) **Graph showing semi-quantitative analysis of infiltrate in H&E sections before and up to 10 days after irradiation. Dotted lines indicate mean + two standard deviations of controls. ●, patients (P); ○, controls (C). No significant differences were present between patients and controls on any time point. **(h) **Inflammatory lesion in a patient with SLE in the vicinity of SBCs 3 days after irradiation. Inflammatory lesions were defined as the presence of category 5 (see Materials and methods) vessel(s) in the dermis, with inflammatory cell infiltration of the epidermal layer coinciding with marked local hydropic degeneration of the basal layer of the epidermis. Insert shows magnification of area with accumulating SBCs. Magnification, ×100. White arrowheads indicate SBCs, and black arrowheads indicate hydropic degeneration.

Additionally, H&E-stained sections from biopsies taken before and after irradiation and biopsies of LE skin lesions were assessed for the presence of inflammatory lesions and co-localisation with SBCs. Inflammatory lesions were defined as the presence of category 5 (see above) vessel(s) in the dermis, with inflammatory cell infiltration of the epidermal layer coinciding with marked local hydropic degeneration of the basal layer of the epidermis (Figure 2h). Co-localisation was regarded as present when inflammatory lesions coincided with the presence of two or more SBCs in the epidermal layer lying over the infiltrate. Scoring was performed by two independent observers not informed about the clinical findings.

### Deposition of Igs and complement

Sequential frozen sections were used for direct immunofluorescent staining of IgM, IgG, IgA, C3c, and C1q, using standard procedures. In brief, sections were washed with PBS and subsequently incubated with the various FITC-labeled specific antibodies at the dilutions indicated. After incubation, sections were washed in PBS again, and nuclei were stained using bisbenzimide (SERVA Electrophoresis GmbH, Heidelberg, Germany) and mounted. Sections were scored by two independent observers for staining at the dermal-epidermal junction (lupus-band) and in the epidermal layer.

### Statistics

Differences between groups were determined using the Mann-Whitney test. The χ^2 ^test was used to analyse categorical variables. Comparison of multiple groups was performed by one-way analysis of variance (Kruskal-Wallis). Correlations between numbers of apoptotic cells detected by H&E staining and numbers detected by cleaved caspase-3 staining and between SBCs and pyknotic nuclear debris were analysed using the nonparametric (Spearman) correlation test. To analyse differences in the level of correlation between patients and controls, slopes of linear regression lines were compared (GraphPad Software 3.02; GraphPad Software, Inc., San Diego, CA, USA).

## Results

### Clearance of apoptotic cells from SLE skin after a single dose of UVB

To investigate apoptotic cell clearance, apoptotic cells were quantified in H&E staining by their altered morphology as SBCs and by the detection of cleaved caspase-3 as a specific apoptotic marker. Significant aspecific staining was observed in basal and spinotic epidermal layers and, to a lesser extent, in the dermis of non-irradiated skin using the TUNEL (terminal deoxynucleotidyl transferase-mediated *in situ *nick-end labeling) detection method (data not shown). However, results from the two detection methods indicated above did highly correlate, *r *= 0.91, *p *< 0.0001. One day after irradiation, apoptotic cells were localised mainly in the stratum spinosum (Figure 1a). After 3 days, approximately 70% of SBCs were localised in the stratum granulosum (Figure 1b), and after 10 days, nearly all apoptotic cells were removed from the skin, partially by shedding (Figure 1c). No SBCs or cleaved caspase-3-positive cells were detected in unexposed skin (Figure 1d,e). After 1 day, SBCs could be detected in patients (88.8 ± 51.8 SBCs per mm^2 ^[mean ± SD]) and controls (101.9 ± 68.6 SBCs per mm^2^, *p *= 0.71). At day 3, the number of apoptotic cells was increased three- to nine-fold in patients (358.2 ± 138.8 SBCs per mm^2^) and controls (321.8 ± 127.3 SBCs per mm^2^, *p *= 0.42). Ten days after irradiation, patients (11.0 ± 11.6 SBCs per mm^2^) and controls (6.0 ± 5.6 SBCs per mm^2^) had decreased, but similar, numbers of apoptotic cells, which resided in the epidermis (*p *= 0.41).

Nuclear dust, defined as one whole pyknotic nucleus or a group of pyknotic nuclear fragments (Figure 1f), could be detected after irradiation. The extent of nuclear dust strongly correlated with numbers of SBCs (*r *= 0.91, *p *< 0.0001). Clearance rate of nuclear dust was comparable with clearance of apoptotic cells and did not differ between patients with SLE and controls (data not shown).

### Development of infiltrates and inflammatory lesions in patients with SLE after irradiation and in LE skin lesions

To study the inflammatory response induced by a single dose of UVB irradiation, skin biopsies were taken after 1, 3, and 10 days and stained with H&E. In general, in skin from control subjects, some influx of inflammatory cells was seen after 1 day, decreasing over time with only low influx remaining after 10 days compared with non-irradiated skin. Influx was localised mainly around the dermal blood vessels (Figure 2a–c,g). In five out of 15 patients, influx of cells was increased, especially after 3 days, and persisted after 10 days (Figure 2d–g). In two of these patients, the infiltrate progressed toward the basal layer of the epidermis, which was damaged as indicated by the presence of marked hydropic degeneration. Based on pre-defined criteria, these were considered inflammatory lesions (Materials and methods). Inflammatory lesions were localised only in the vicinity of apoptotic keratinocytes (Figure 2h).

**Figure 3:**
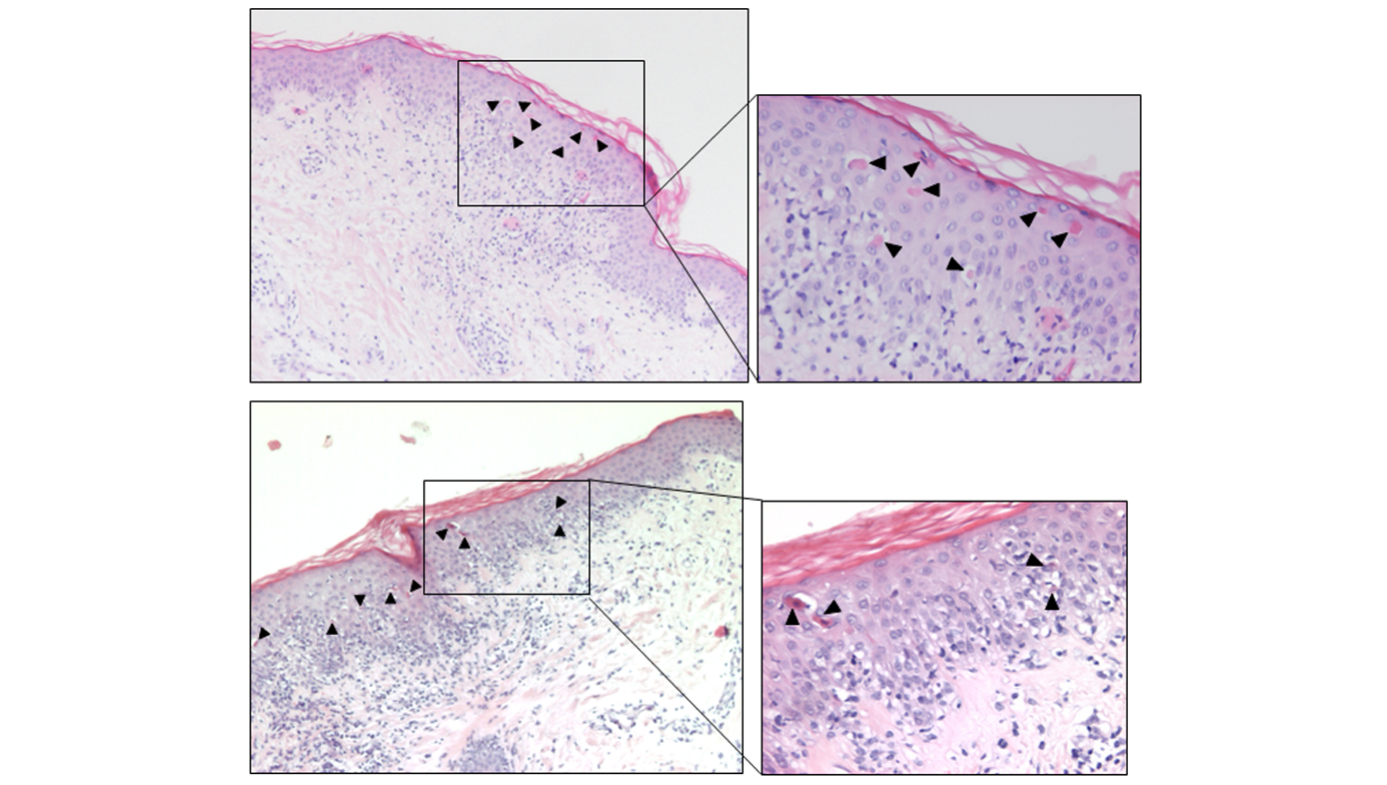
Co-localisation of apoptotic keratinocytes and infiltrate in lupus erythematosus (LE) skin lesions. Sections of two representative LE skin lesions showing an area of local accumulation of apoptotic keratinocytes and local infiltration of inflammatory cells and hydropic degeneration of the epidermis. Arrowheads indicate apoptotic keratinocytes. Magnifications, ×40 (left panels) and ×200 (right panels).

To determine whether the inflammatory lesions seen in the vicinity of SBCs after irradiation might also be present in established LE lesions, biopsies of 20 LE skin lesions were assessed for co-localisation of infiltrate and SBCs (Table 2). In 16 out of 20 LE biopsies, SBCs could be detected, and in 10 of these biopsies, co-localisation of inflammatory lesions and local accumulation of SBCs was seen (Figure 3). The latter group of patients could not be distinguished from the other patients by any of the characteristics depicted in Tables 1 and 2.

### Characterisation and quantification of infiltrating cells and deposition of Igs and complement

**Figure 4:**
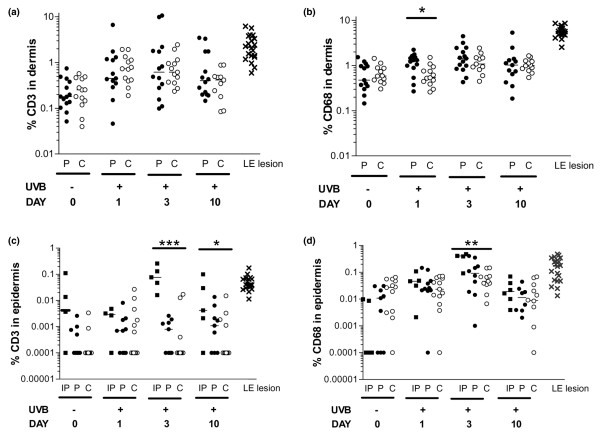
Infiltration of T cells and macrophages into the papillary dermis and epidermis. **(a) **Percentage of CD3 staining in the dermis before and after irradiation in 15 patients with systemic lupus erythematosus (SLE) and 12 controls and in 20 lupus erythematosus (LE) biopsies quantified by morphometry. **(b) **Percentage of CD68 staining in the dermis before and after irradiation in 15 patients with SLE and 12 controls and in 20 LE biopsies quantified by morphometry. **(c) **Percentage of CD3 staining in the epidermis before and after irradiation comparing patients with infiltrates (*n *= 5), patients without infiltrates (*n *= 10), and controls (*n *= 13) and in 20 LE biopsies. **(d) **Percentage of CD68 staining in the epidermis before and after irradiation comparing patients with infiltrates (*n *= 5), patients without infiltrates (*n *= 10), and controls (*n *= 13) and in 20 LE biopsies. Median value is indicated by a horizontal line. ■, patients with infiltrates (IP); ●, patients without infiltrates (P); ○, controls (C). *p < 0.05, **p < 0.01, ***p ≤ 0.001. UVB, ultraviolet B light.

To characterise and quantify the infiltrating cells, sections were stained using a T-cell (CD3) and monocyte/macrophage (CD68) marker. Subsequently, staining in the papillary dermis and epidermis was quantified by morphometry. In the dermis of non-irradiated skin, low numbers of T cells (0.25% ± 0.20% in patients versus 0.27% ± 0.17% in controls) and macrophages (0.69% ± 0.45% in patients versus 0.70% ± 0.33% in controls) were present. Influx of T cells and macrophages increased in all subjects 1 day after irradiation, declined slowly, and was only slightly increased after 10 days compared with non-irradiated skin (Figure 4a,b). Macrophages were increased in the skin of patients with SLE after 1 day as compared with controls (1.19% ± 0.55% and 0.66% ± 0.35%, respectively, *p *= 0.02). T-cell influx was not significantly different at any time point between patients and controls. Neutrophils were accidentally (one to five cells per 4 mm section) detected in H&E staining in both controls and patients (data not shown). Five patients developed infiltrates of inflammatory cells detected by semi-quantitative analysis in H&E-stained sections and by morphometric analysis in CD3- or CD68-stained sections on at least two different days after irradiation. This group of patients could not be distinguished from the other patients by any of the patient characteristics listed in Tables 1 and 2. In the patients who developed infiltrates, levels of T-cell and macrophage influx were in the same range as seen in 20 skin biopsies from patients with cutaneous LE lesions (Figure 4). This group of patients with cutaneous LE lesions could not be distinguished from the patients who got UVB irradiation to the skin by any of the patient characteristics listed in Tables 1 and 2.

Influx of T cells and macrophages was not limited to the dermal layer of the skin. T cells and, especially, macrophages were detected in the epidermis as well. In the epidermis of non-irradiated skin, almost no T cells (0.010% ± 0.011% in patients, 0.028% ± 0.023% in controls) (Figure 4c) or macrophages (0.026% ± 0.047% in patients, 0.0005% ± 0.0008% in controls) (Figure 4e) could be detected. However, 3 days after irradiation, a substantial number of macrophages were observed in the epidermal compartment. Influx into the epidermis was higher in patients (0.038% ± 0.074% for CD3, *p *= 0.02, and 0.26 ± 0.31 for CD68, *p *= 0.04) compared with controls (0.0023% ± 0.0057% for CD3 and 0.061% ± 0.046% for CD68). Furthermore, epidermal influx was higher in patients with infiltrates compared with patients without these infiltrates and controls (*p *= 0.0009 for CD3-positive T cells and *p *= 0.009 for CD68-positive macrophages) (Figure 4d,f).

Deposition of Igs and complement factors was studied to assess their potential involvement in the inflammatory response. Most intense staining of Igs and complement in the epidermal layer was seen near local accumulations of epidermal apoptotic cells. However, depositions were not restricted to patients and could also be detected in all healthy controls (data not shown).

### Rate of clearance of apoptotic cells in patients with infiltrates

**Figure 5:**
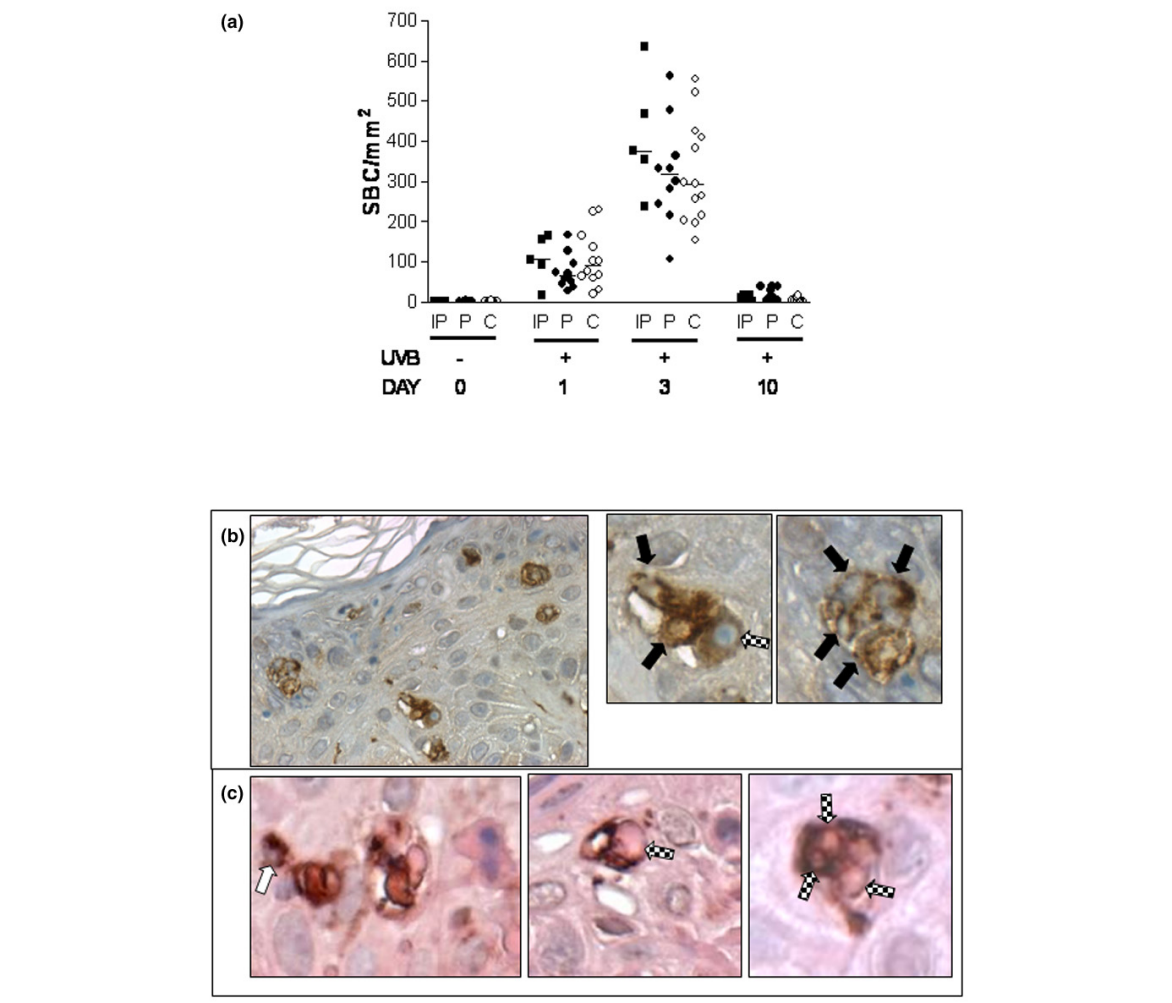
Presence of apoptotic cells and phagocytosis by macrophages comparing controls and patients with or without infiltrates. **(a) **Numbers of sunburn cells (SBCs) in patients with infiltrates, patients without infiltrates, and controls before and up to 10 days after irradiation. ■, patients with infiltrates (IP); ●, patients without infiltrates (P); ○, controls (C). Extensive phagocytosis of apoptotic keratinocytes by macrophages in the epidermis of patients with infiltrates. **(b) **Representative biopsy showing CD68 staining combined with haematoxylin staining using diaminobenzidine (DAB) for visualisation of CD68-positive cells. Magnification, ×400. Two macrophages that contain multiple phagocytic vacuoles (black arrows) are shown. One vacuole clearly contains an apoptotic cell (checkered arrow). **(c) **Representative biopsy showing CD68 staining using DAB for visualisation, combined with haematoxylin eosin staining. Magnification, ×400. White arrow indicates macrophages not involved in phagocytosis, and checkered arrows indicate eosinophilic particles that are being ingested by macrophages. UVB, ultraviolet B light.

Numbers of apoptotic cells were compared between patients with and without infiltrates to evaluate whether differences in clearance rate of apoptotic cells might have been responsible for the development of infiltrates. Patients with infiltrates in the skin did not have increased numbers of apoptotic cells at any time point compared with patients without infiltrates and controls (Figure 5a). Also, extent of nuclear dust did not differ at any time point between patients with infiltrates and the remaining patients without these infiltrates and controls (data not shown).

### Phagocytosis of apoptotic keratinocytes by macrophages in the epidermis

Macrophages in the epidermis were often localised in the vicinity of apoptotic cells. Only in two patients with infiltrates, a large proportion of the epidermal macrophages contained multiple large vacuoles (Figure 5b). The morphology of these vacuoles indicated ingestion of apoptotic cells. This was confirmed by counterstaining with H&E which showed that the apoptotic bodies ingested by macrophages had an eosinophilic stained cytoplasm (Figure 5c).

## Discussion

In the present study, we demonstrated that the rate of clearance of apoptotic cells after a single standardised dose of UVB is not decreased in the skin of patients with SLE. However, we showed that in a subset of patients with SLE, UVB irradiation results in the development of infiltrates and inflammatory lesions in the vicinity of apoptotic cells. Furthermore, co-localisation of inflammatory lesions and apoptotic cells was frequently seen in LE skin lesions, suggesting that inflammation after a single dose of UVB might represent early LE skin lesions in which apoptotic cells play an inducing role. The infiltrate that developed after irradiation consisted mainly of T cells and macrophages and was localised in the dermis and epidermis. In some patients with infiltrates, epidermal macrophages were phagocytosing multiple apoptotic cell bodies.

The clearance rate of apoptotic cells or nuclear debris did not differ between patients with SLE and controls. Also, the clearance rate of apoptotic cells in patients with infiltrates was not different from that of patients without these infiltrates and controls. These data contrast with a recent paper by Kuhn *et al*. [22] showing that apoptotic cell clearance after UV irradiation was defective in the skin of patients with non-systemic cutaneous LE. Differences in methods used to detect apoptotic cells might, at least in part, explain these discrepancies. Kuhn *et al*. used two nick-labeling techniques to detect apoptosis. As discussed in a recent commentary [23], detection of DNA nicks after UVB irradiation is not a reliable measure of apoptosis [24-26]. We used other methods for detection of apoptotic cells, namely by morphology in H&E-stained sections and by specific staining for the cleaved form of caspase-3. Detection of SBCs by H&E staining has been used extensively for many years and detects apoptotic cells at a relatively late stage in the apoptotic process [14,27]. Cleaved caspase-3 is an early marker for apoptosis; its activity precedes all the morphological changes that are initiated [28]. Both methods correlated well.

*In vitro*, several reports have described decreased uptake of apoptotic cells in SLE [8,29]. We recently showed that macrophages of patients with SLE do not have intrinsic defects in the clearing of apoptotic cells. However, decreased levels of complement such as C1q and C4, during active disease in particular, may lead to decreased phagocytosis of apoptotic cells [10]. Also, experiments in lupus-prone mouse strains suggest that the capacity of macrophages to internalise apoptotic cells is normal when mice are not in a state of active disease [30,31]. Our data provide *in vivo *evidence that the apoptotic cell clearance rate in patients with inactive SLE is not disturbed in the skin after a single UVB exposure.

Clearance of apoptotic cells by phagocytes is usually an anti-inflammatory event. We demonstrated that T cells and mainly macrophages infiltrate into the epidermal layers of the skin. This influx was almost restricted to patients and occurred significantly more frequently in patients with infiltrates in the dermal layer. Epidermal macrophages were often seen in the vicinity of apoptotic cells and sporadically showed single ingested apoptotic bodies. Macrophages in the epidermis of two patients with infiltrates were phagocytosing multiple apoptotic keratinocytes. To our knowledge, this phenomenon has not been described in the skin before. These data suggest that, in controls, the major part of apoptotic cells are cleared by shedding and, possibly, phagocytosis by keratinocytes [32], whereas only low numbers of macrophages seem to be involved in the clearance of apoptotic cells from the skin. The association between formation of infiltrates and participation of macrophages in apoptotic cell clearance suggests a pro-inflammatory role of the macrophages in these patients.

We hypothesise that the development of infiltrates as seen in patients with SLE could be the result of inflammatory clearance of apoptotic cells. Autoantibodies, especially directed against SSA/Ro, have been shown to bind to apoptotic keratinocytes. Other reports have shown that binding of anti-phospholipid antibodies to apoptotic cells results in increased production of the pro-inflammatory cytokine tumour necrosis factor-α [33]. Furthermore, Meller *et al*. [34] reported that UV light-induced injury leads to apoptosis, necrosis, and cytokine/chemokine production that results in an amplification cycle leading to cutaneous LE lesions. However, they failed to determine which factor specifically induces inflammation in LE lesions. Binding of autoantibodies to apoptotic keratinocytes in the skin and subsequent pro-inflammatory clearance by macrophages could be involved in induction of infiltrates in our study. No association was found, however, between any particular autoantibody specificity and the occurrence of infiltrates, although this could be due to the limited group size. This hypothesis, however, is supported by a very recent article by Clancy *et al*. [35] showing that maternal autoantibodies may bind apoptotic cardiomyocytes and promote inflammation thereby contributing to the pathogenesis of congenital heart block.

It can be argued that our study has several other limitations as well. First of all, these studies were conducted in only a limited number of subjects and caution should be taken extrapolating these results to the whole SLE population. Second, although we used the MED to correct for differences in UV sensitivity, inter-individual variation in skin types might have influenced the results. Furthermore, given that erythema can be seen as an early inflammatory response mediated largely by prostaglandins produced in the skin, any influence of immunosuppressive medication used cannot be excluded [36]. Such an effect, however, seems unlikely. Because topical administration of corticosteroids can influence the MED response [37], patients using topical corticosteroids at the time of the study were excluded. Systemic administration of corticosteroids up to 80 mg daily was reported not to decrease erythema [38]. Consequently, corticosteroids that were used by our patients at substantially lower doses (Tables 1 and 2) have in all likelihood not influenced our results. Third, several reports have suggested that multiple UVB exposures induce macroscopic LE-like lesions [39,40]. Wolska *et al*. [41] have shown that single exposure also can result in macroscopic lesions. We deliberately chose a single-exposure approach because this method has several advantages compared with the multiple-exposure approach. Most importantly, single exposure enables the analysis of stimulus and effect (that is, apoptosis induction, apoptotic cell clearance, and induction of inflammation) without interference of processes induced by repeated exposures. Infiltrates were most apparent 3 and 10 days after a single standardised dose of UVB. Although erythema was still visible after 3 days, no clear macroscopic lesions could be detected at that time, probably due to the small size of the irradiated areas. The irradiated areas were intentionally kept small because of the potential hazard of activating systemic disease by irradiating large skin areas in patients with SLE.

## Conclusion

From our data, we conclude that UVB irradiation of the skin of patients with SLE can induce infiltrates and inflammatory lesions. Although *in vivo *clearance of apoptotic cells after UVB irradiation in patients with SLE was normal, the development of lesions in the vicinity of apoptotic cells and their association with increased phagocytic activity of macrophages suggest that an altered, inflammatory clearance of apoptotic cells might be important in the development of lupus skin lesions.

## Abbreviations

BSA = bovine serum albumin; DAB = diaminobenzidine; EDTA = ethylenediaminetetraacetic acid; FcγR = Fc gamma receptor; FITC = fluorescein isothiocyanate; H&E = haematoxylin eosin; HRP = horseradish peroxidase; Ig = immunoglobulin; MED = minimal erythemal dose; PBS = phosphate-buffered saline; SBC = sunburn cell; SD = standard deviation; SLE = systemic lupus erythematosus; UVB = ultraviolet B light.

## Competing interests

The authors declare that they have no competing interests.

## Authors' contributions

ER participated in the design of the study, performed the experimental work, and wrote and rewrote all versions of the manuscript. MdJ participated in the design, execution, and analysis of the IgG and complement deposition. HK assisted in interpreting the histopathological pictures. MFJ participated in the set-up of the study and helped draft the manuscript. PL participated in the set-up of the study and analysis of the results and helped draft the manuscript. CK participated in the set-up of the study and helped draft the manuscript. MB participated in the set-up of the study and analysis of the results and helped draft the manuscript. All authors read and approved the final manuscript.

## Figures and Tables

**Table 1:** Characteristics of patients included in the UVB irradiation study: cumulative ACR criteria, autoantibody specificities, medication, and MED at the time of the study

Patient characteristics																			
Infiltrate	No.	Gender	Age					A	C	R					Prednisone	Hydroxychloroquine	Azathio prine	Autoantibody	MED
			(years)	1	2	3	4	5	6	7	8	9	10	11	(mg/day)	(mg/day)	(mg/day)	specificities	(J/cm^2^)

+	1	F	34	+	-	-	-	+	-	-	-	+	+	+	3.75	400	-	SSA/nRNP/dsDNA/Sm/phosL	0.090
-	2	F	64	-	+	-	-	-	-	-	-	+	+	+	10	-	100	dsDNA/SSA/phosL	0.280
-	3	F	33	+	-	-	-	+	+	+	-	+	+	+	5	400	100	dsDNA	0.075
-	4	F	53	+	-	+	-	+	+	+	-	+	+	+	5	-	75	dsDNA	0.070
-	5	F	36	-	-	-	-	+	+	-	-	+	+	+	-	600	-	dsDNA/nRNP	0.100
+	6	M	76	-	-	-	-	+	+	+	+	+	+	+	7.5	-	-	dsDNA/SSA	0.090
-	7	M	38	+	-	-	-	-	-	-	-	-	-	-	5	-	75	dsDNA/SSA/SSB	0.050
+	8	F	64	-	-	-	-	+	+	-	-	+	+	+	-	-	150	dsDNA	0.180
-	9	F	50	-	-	-	-	+	-	+	-	+	+	+	10	-	100	dsDNA/SSA	0.180
-	10	M	67	-	-	+	-	-	-	+	-	+	-	+	3.5	-	25	SSA/phosL	0.070
-	11	F	42	-	-	+	+	+	-	-	-	-	+	+	5	400	75	dsDNA/SSA/nRNP/Sm	0.130
+	12	M	49	-	-	+	-	+	+	-	-	-	-	+	5	-	-	nRNP	0.100
-	13	F	57	-	+	-	-	+	-	+	-	+	+	+	5	-	100	dsDNA/Sm	0.085
+	14	F	53	-	-	+	-	+	-	-	-	-	+	+	-	200	-	dsDNA/nRNP	0.075
-	15	F	47	+	+	+	-	-	-	+	-	+	-	+	5	-	150	SSA/phosL	0.075

ACR criteria numbered according to Bombardier *et al*. [19]: 1, malar (or 'butterfly') rash; 2, discoid rash; 3, sensitivity to light, or photosensitivity; 4, oral ulcers; 5, arthritis; 6, serositis; 7, kidney disorder; 8, neurologic disorder; 9, blood abnormalities; 10, positive antiphospholipid antibody test; 11, immunologic disorder, including lupus anticoagulant, positive anti-double-stranded DNA, false-positive syphilis test, or positive anti-Smith test (such as anticardiolipin). + and - indicate cumulative presence or absence, respectively, of a particular criterion. In the leftmost column, + indicates patients with infiltrates. ACR, American College of Rheumatology; F, female, M, male; MED, minimal erythemal dose; UVB, ultraviolet B light.

**Table 2:** Characteristics of patients included in the LE skin biopsies: cumulative ACR criteria, autoantibody specificities, and medication

Patient characteristics																		
SBC/co-loc	No.	Gender	Age					A	C	R					Prednisone	Hydroxychloroquine	Azathioprine	Autoantibody
			(Years)	1	2	3	4	5	6	7	8	9	10	11	(mg/day)	(mg/day)	(mg/day)	specificities

+/+	1	F	45	-	-	-	-	-	+	+	-	-	+	+		-	75	SSA/SSB/dsDNA
+/+	2	F	51	+	-	+	-	-	+	-	-	+	-	+	5	-	100	SSA/SSB
+/+	3	F	70	-	+	-	-	-	-	-	-	+	+	+	10	200	25	Sm/SSA/nRNP
-/-	4	M	47	-	+	+	-	-	-	-	-	+		+	-	-	-	SSA/SSB
-/-	5	F	45	+	+	-	+	+	-	-	-	+	+	+	-	5	-	Sm/nRNP/CL
+/-	6	F	50	+	+	-	+	+	-	-	-	+	+	+	12.5	600	-	Sm/nRNP/CL
+/+	7	F	49	+	+	-	+	+	-	-	-	+	+	+	10	600	-	Sm/nRNP/CL
+/-	8	F	39	+	-	+	+	+	-	-	-	-	+	+	60	-	-	dsDNA
+/+	9	F	41	+	-	-	-	+	+	-	-	-	+	+	2.5	-	75	SSA/SSB/dsDNA
+/+	10	F	45	-	+	-	-	+	+	+	-	+	+	+	5	400	-	dsDNA
+/+	11	F	46	-	+	-	-	+	+	+	-	+	+	+	5	400	-	dsDNA
+/-	12	F	38	-	+	-	-	+	-	-	-	-	+	+	-	-	-	dsDNA
-/-	13	F	75	-	-	-	-	+	-	-	-	+	+	+	-	-	-	dsDNA/CL
+/-	14	F	33	+	-	-	-	+	-	-	-	+	+	+	-	-	-	SSA/Sm/nRNP/dsDNA
+/+	15	F	58	-	+	+	-	-	-	-	-	+	+	+	-	-	-	SSA/dsDNA
+/-	16	F	38	-	+	+	-	-	-	-	-	+	+	+	-	-	-	SSA/dsDNA
-/-	17	F	49	-	+	+	-	-	-	+	-	+	-	+	-	800	-	dsDNA/CL
+/+	18	F	49	-	+	-	+	+	+	-	-	+	+	+	7.5	-	-	dsDNA
+/+	19	M	35	-	+	+	-	-	-	-	-		+	+	-	-	-	SSA/dsDNA
+/-	20	F	26	+	-	+	-	-	-	-	-	+	+	+	-	-	-	nRNP/Sm/dsDNA

ACR criteria numbered according to Bombardier *et al*. [19]: 1, malar (or 'butterfly') rash; 2, discoid rash; 3, sensitivity to light, or photosensitivity; 4, oral ulcers; 5, arthritis; 6, serositis; 7, kidney disorder; 8, neurologic disorder; 9, blood abnormalities; 10, positive antiphospholipid antibody test; 11, immunologic disorder, including lupus anticoagulant, positive anti-double-stranded DNA, false-positive syphilis test, or positive anti-Smith test (such as anticardiolipin). + and - indicate cumulative presence or absence, respectively, of a particular criterion. In the leftmost column, **+/- **indicates biopsies containing SBCs but no apparent co-localisation, **+/+ **indicates biopsies containing co-localisation of inflammatory lesions and SBCs, and **- **indicates biopsies without SBCs. ACR, American College of Rheumatology; co-loc, co-localisation; F, female, LE, lupus erythematosus; M, male; SBC, sunburn cell; UVB, ultraviolet B light.
